# Vitamin D deficiency causes insulin resistance by provoking oxidative stress in hepatocytes

**DOI:** 10.18632/oncotarget.18754

**Published:** 2017-06-28

**Authors:** Sha Tao, Qi Yuan, Li Mao, Feng-Li Chen, Feng Ji, Zhao-Hui Cui

**Affiliations:** ^1^ Department of Endocrinology, Huai’an First People's Hospital, Nanjing Medical University, Huai’an, China; ^2^ Clinical Laboratory, Huai’an First People's Hospital, Nanjing Medical University, Huai’an, China; ^3^ Department of Orthopedics, Huai’an First People's Hospital, Nanjing Medical University, Huai’an, China

**Keywords:** vitamin D, insulin resistance, oxidative stress, hepatocytes, N-acetylcysteine (NAC)

## Abstract

Vitamin D deficiency could cause insulin resistance. However, the underlying mechanisms are unclear. The 1α-Hydroxylase [“1α(OH)ase”] is a key enzyme for activate vitamin D3 synthesis. Here, we show that 1α(OH)ase stable knockdown by targeted shRNA led to vitamin D3 depletion in L02 hepatocytes. 1α(OH)ase silence also inhibited insulin-induced downstream signaling (IRS-1, ERK and AKT) transduction and glucose transporter 4 expression. Further, 1α(OH)ase shRNA in L02 hepatocytes led to significant reactive oxygen species production, p53-p21 activation and DNA damages. Such effects were almost completely reversed with co-treatment of n-acetylcysteine, which is an established anti-oxidant. Remarkably, insulin-induced downstream signaling transduction and glucose transporter 4 expression were recovered with n-acetylcysteine co-treatment in 1α(OH)ase-silenced L02 hepatocytes. Together, our results suggest that vitamin D deficiency-induced insulin resistance is possibly caused by oxidative stress in hepatocytes.

## INTRODUCTION

Insulin resistance is a major reason of type-II diabetes [1, 2]. It is also a characteristic manifestation of a wide range of other clinical diseases [3–5]. A number of genetic and/or environmental factors could cause insulin resistance [3–5]. Epidemic studies have suggested that that vitamin D deficiency is also associated with insulin resistance [6]. The supplementation of active vitamin D3 may help to improve the insulin resistance [6]. However, the underlying mechanism is largely unknown [6].

The function of reactive oxygen species (ROS) in insulin resistance has been well established [7–9]. Studies have demonstrated that ROS level is significantly increased in both clinical samples and experimental settings of insulin resistance [10–12]. Meanwhile, exogenous oxidative stress would lead to insulin resistance. These results suggest that oxidative stress might play a key function in insulin resistance [7, 9]. Inhibition of ROS, on the other hand, could improve insulin sensitivity and glucose homeostasis in insulin-resistant mice [10–12]. Clinical studies have also shown that supplement with anti-oxidant may improve insulin sensitivity [10–12].

25-Hydroxyvitamin D3 1α-Hydroxylase [“1α(OH)ase”] is a key enzyme for activate vitamin D3 synthesis [13, 14]. In the current study, 1α(OH)ase was silenced in human L02 hepatocytes to mimic vitamin D deficiency. Our results suggest that vitamin D deficiency induces insulin resistance probably by provoking oxidative stress.

## RESULTS

### Knockdown of 1α(OH)ase leads to vitamin D3 depletion in L02 hepatocytes

**Figure 1:**
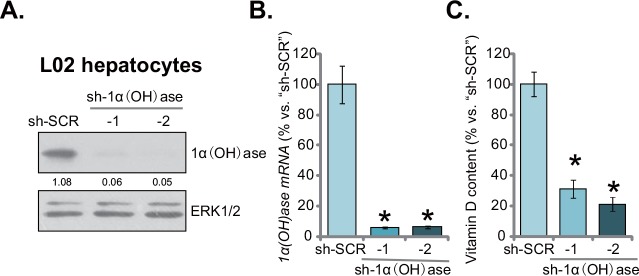
Knockdown of 1α(OH)ase leads to vitamin D3 depletion in L02 hepatocytes Puromycin-selected stable L02 hepatocytes, expressing shRNA against human 1α-Hydroxylase [“sh-1α(OH)ase-1/-2”] or the scramble control shRNA (“sh-SCR”), were subjected to Western blotting assay **(A)** and qRT-PCR assay **(B)** to test 1α(OH)ase expression; Vitamin D3 content in the conditional medium was also analyzed **(C)**. Relative 1α(OH)ase expression (vs. loading control ERK1/2) was quantified (A). Data were expressed as mean ± SD (n=5). * *p* <0.05 *vs.* “sh-SCR” cells. Experiments in this and all following figures were repeated three times, and similar results were obtained.

In order to mimic vitamin D deficiency *in vitro*, shRNA strategy was applied to knockdown vitamin D3 1α-Hydroxylase [“1α(OH)ase”] in human L02 hepatocytes. 1α(OH)ase is required for vitamin D3 synthesis [13]. As described, two distinct lentiviral shRNAs (“-1/-2”), targeting non-overlapping sequences of human 1α(OH)ase, were applied. Western blotting assay results showed that stable introduction (via lentiviral infection) of either shRNA led to dramatic downregulation of 1α(OH)ase protein in L02 hepatocytes (Figure 1A). Meanwhile, *1α(OH)ase mRNA* was almost completely depleted by the targeted shRNA (Figure 1B). Consequently, the cellular content of vitamin D3 was dramatically reduced in 1α(OH)ase-silenced L02 hepatocytes (Figure 1C). Notably, the scramble control shRNA (“sh-SCR”) had no significant effect on 1α(OH)ase expression nor vitamin D3 content. Together, these results demonstrate that 1α(OH)ase knockdown by targeted shRNAs leads to vitamin D3 depletion in human L02 hepatocytes.

### Knockdown of 1α(OH)ase leads to insulin resistance in L02 hepatocytes

**Figure 2:**
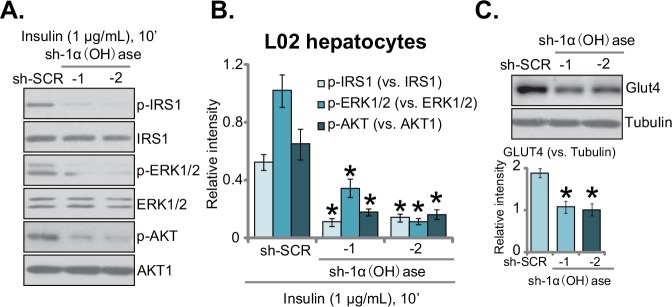
Knockdown of 1α(OH)ase leads to insulin resistance in L02 hepatocytes Puromycin-selected stable L02 hepatocytes, expressing shRNA against human 1α-Hydroxylase [“sh-1α(OH)ase-1/-2”] or the scramble control shRNA (“sh-SCR”), were treated with insulin (1 μg/mL) for 10 min, expressions of listed proteins were tested by Western blotting assay **(A)**; quantified results summarizing three sets of repeated blot data were also shown **(B)**; expressions of GLUT4 and (β-) tubulin were also tested, results were also quantified **(C)**. Data were expressed as mean ± SD (n=3). * *p* <0.05 *vs.* “sh-SCR” cells.

The aim of this study is to test the potential effect of vitamin D deficiency on insulin resistance. The 1α(OH)ase-silenced L02 hepatocytes (See Figure 1) were thereby treated with insulin. Western blotting assay was performed to test insulin signalings [15, 16]. Results in Figure 2A demonstrated that insulin (1 μg/mL, 10 min)-induced activation of downstream signalings, including IRS-1 (insulin receptor substrate-1), ERK1/2 and AKT, was largely inhibited with 1α(OH)ase knockdown. Phosphorylated (“p-”) IRS-1, p-AKT and p-ERK1/2 by insulin were all dramatically reduced in 1α(OH)ase-depleted L02 hepatocytes (Figure 2A). Expression of above total kinases was yet unchanged (Figure 2A). Quantified results summarizing three sets of repeated blot data further confirmed inhibition of insulin-induced IRS-1, ERK1/2 and AKT activations with 1α(OH)ase silence in L02 hepatocytes (Figure 2B). Meanwhile, as shown in Figure 2C, expression of glucose transporter 4 (GLUT4), a key glucose transporter protein, was also downregulated in 1α(OH)ase-silenced L02 hepatocytes (Figure 2C). Together, these results imply that knockdown of 1α(OH)ase might lead to insulin resistance in L02 hepatocytes.

### Knockdown of 1α(OH)ase leads to ROS production, p53-p21 activation and DNA damage in L02 hepatocytes

**Figure 3:**
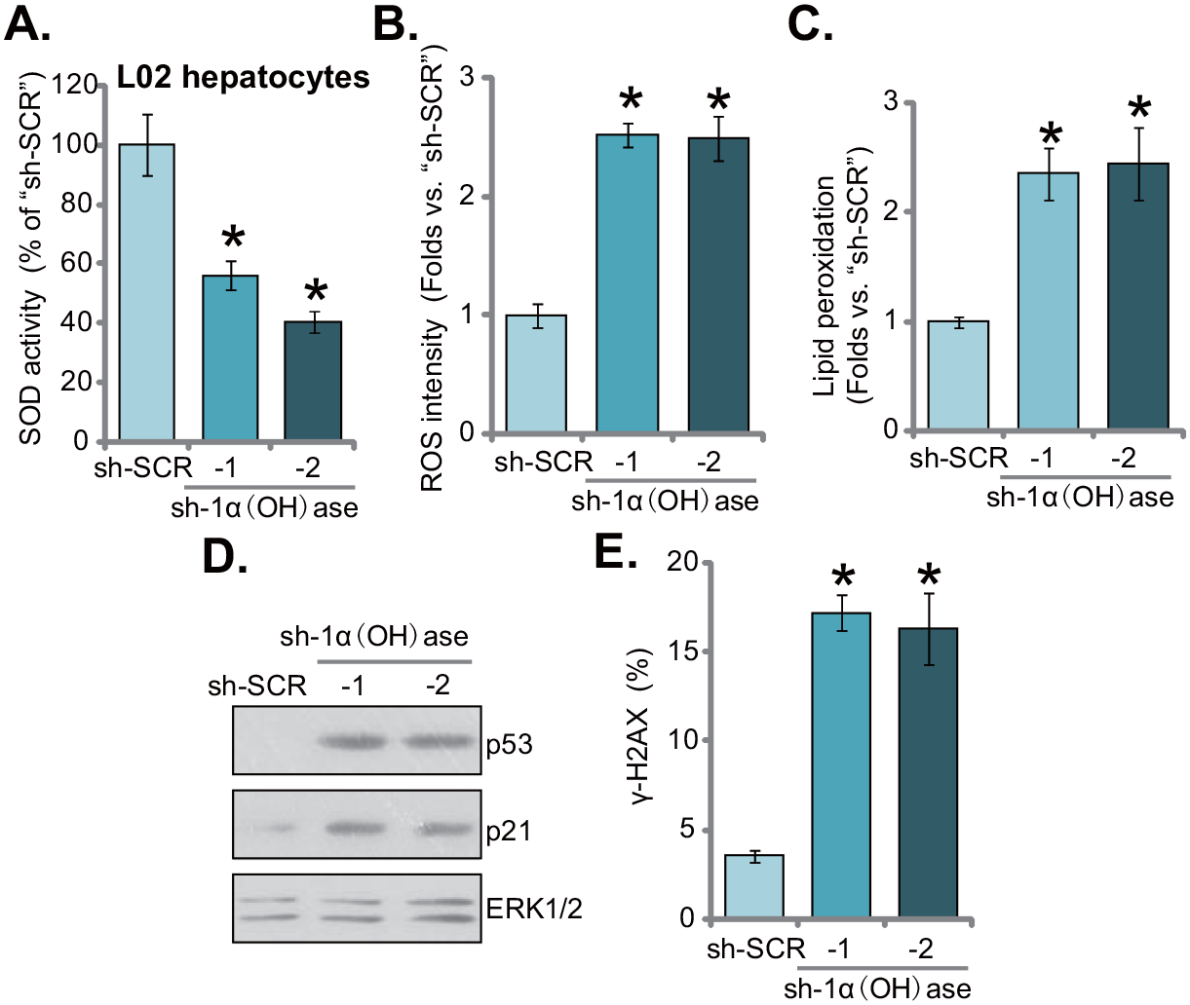
Knockdown of 1α(OH)ase leads to ROS production, p53-p21 activation and DNA damage in L02 hepatocytes Puromycin-selected stable L02 hepatocytes, expressing shRNA against human 1α-Hydroxylase [“sh-1α(OH)ase-1/-2”] or the scramble control shRNA (“sh-SCR”), were subjected to listed assays to test SOD activity **(A)**, ROS content (DCFH-DA assay) **(B)**, lipid peroxidation level (TBAR assay) **(C)**, p53-p21 signaling (Western blotting assay) **(D)**, and DNA damage (γ-H2AX FACS assay) **(E)**. Data were expressed as mean ± SD (n=5). * *p* <0.05 *vs.* “sh-SCR” cells.

It has been previously shown that vitamin D and 1α(OH)ase are both involved in prevention of oxidative stress [17–19]. Vitamin D activates vitamin D receptor (VDR) to increase activity of superoxide dismutase (SOD), phospholipid hydroperoxide glutathione peroxidase (GSH-Px) and other anti-oxidant enzymes [19], thereby suppressing oxidative stresses [19]. Further, ROS production could be an important contributor of insulin resistance [8, 9]. Here, we found that SOD activity was also significantly decreased in 1α(OH)ase-silenced L02 hepatocytes (Figure 3A). Consequently, cellular ROS content and subsequent lipid peroxidation intensity were both dramatically increased (Figure 3B and 3C). Thus, 1α(OH)ase knockdown apparently provoked oxidative stress in human L02 hepatocytes (Figure 3B and 3C). Further studies showed that 1α(OH)ase silence in L02 hepatocytes also activated p53-p21 signaling (Figure 3D), which is a key downstream pathway following oxidative stress [20, 21]. Further, level of DNA damage, tested by γ-H2AX FACS assay, was also increased in L02 hepatocytes expressing 1α(OH)ase shRNAs (Figure 3E).

### N-acetylcysteine blocks ROS production, p53-p21 activation and DNA damage in 1α(OH)ase-silenced L02 hepatocytes

**Figure 4:**
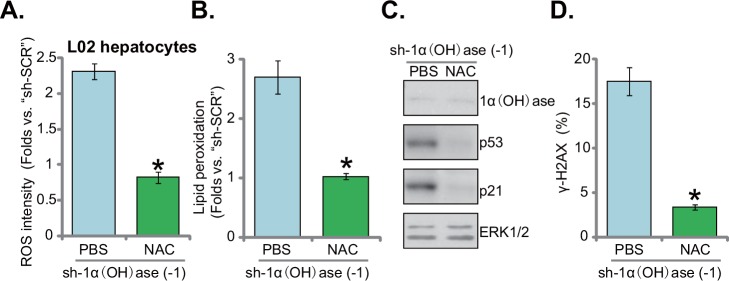
N-acetylcysteine blocks ROS production, p53-p21 activation and DNA damage in 1α(OH)ase-silenced L02 hepatocytes L02 hepatocytes, infected with shRNA against human 1α-Hydroxylase [“sh-1α(OH)ase-1”], were also exposed to n-acetylcysteine (NAC, 500 μM, renewed daily) or PBS. After 10 days, relative ROS content (DCFH-DA assay) **(A)**, lipid peroxidation level (TBAR assay) **(B)** p53-p21 signaling (Western blotting assay) **(C)**, and DNA damage (γ-H2AX FACS assay) **(D)** were tested. Data were expressed as mean ± SD (n=5). * *p* <0.05 *vs.* “PBS” cells.

To study the potential effect of oxidative stress on insulin resistance in 1α(OH)ase-depleted L02 hepatocytes, the well-established anti-oxidant n-acetylcysteine (NAC) [22, 23] was applied. Results in Figure 4A confirmed that co-treatment with NAC almost completely blocked oxidative stress in 1α(OH)ase-silenced L02 hepatocytes (by “shRNA-1”, see Figure 1). ROS level reduced to even lower than control level with NAC co-administration (Figure 4A). Consequently, lipid peroxidation by 1α(OH)ase shRNA was almost completely nullified by NAC (Figure 4B). p53-p21 activation in 1α(OH)ase-silenced cells was also blocked (Figure 4C). Similarly, 1α(OH)ase silence-induced DNA damage, tested again by the γ-H2AX FACS assay (Figure 4D), was also significantly alleviated with co-treatment of NAC. As expected, NAC co-treatment failed to rescue 1α(OH)ase expression in L02 hepatocytes (Figure 4C). Together, these results indicate that co-treatment with NAC almost blocked 1α(OH)ase depletion-induced ROS production, p53-p21 activation and DNA damage in L02 hepatocytes.

### NAC restores insulin sensitivity in 1α(OH)ase-silenced L02 hepatocytes

**Figure 5:**
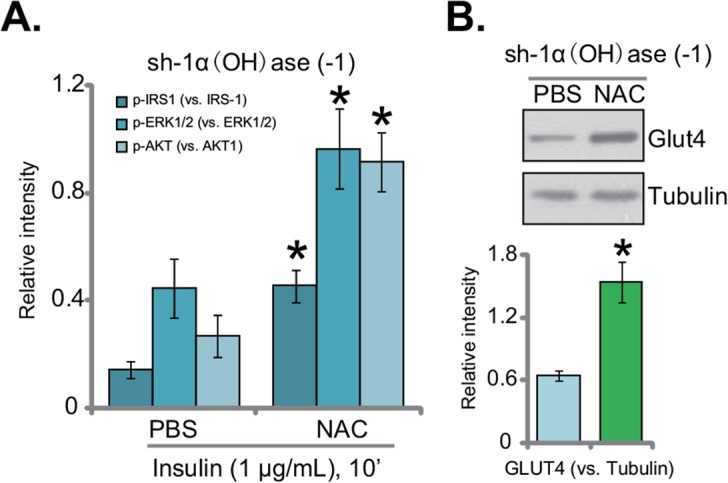
NAC restores insulin sensitivity in 1α(OH)ase-silenced L02 hepatocytes L02 hepatocytes, infected with shRNA against human 1α-Hydroxylase [“sh-1α(OH)ase-1”], were also exposed to n-acetylcysteine (NAC, 500 μM, renewed daily) or PBS; After 10 days, cells were treated with insulin (1 μg/mL) for 10 min, expressions of listed proteins were tested by Western blotting assay, and results of three sets of repeats were quantified **(A)**; expressions of GLUT4 and tubulin were also tested. **(B)** Quantified results summarizing three sets of repeated blot data were also shown). Data were expressed as mean ± SD (n=3). * *p* <0.05 *vs.* “PBS” cells.

Oxidative stress is proposed as the major cause of insulin resistance [7]. If ROS production is the reason of insulin resistance in 1α(OH)ase-silenced hepatocytes, inhibition of ROS by NAC (see Figure 4) should restore insulin sensitivity. Indeed, as shown in Figure 5A (quantified blot results), insulin-induced downstream signaling activation was recovered with NAC co-treatment in the 1α(OH)ase-silenced cells. In 1α(OH)ase-silenced cells, after co-treatment of NAC, activations of IRS-1, AKT and ERK1/2 by insulin returned to control level (“sh-SCR”) (Figure 5A, quantified blot results). Furthermore, downregulation of GLUT4 by 1α(OH)ase silence was also reversed with NAC co-treatment (Figure 5B). Thus, these results indicate that oxidative stress should be the reason of insulin resistance in 1α(OH)ase-silenced cells, which was reversed with co-treatment of NAC.

## DISCUSSION

Vitamin D is a key hormone that is vital in the regulation of mineral homeostasis. It is mainly involved in bone and calcium/phosphorus balance. Recent studies have implied its function in the pathogenesis of insulin resistance and type II diabetes [6, 24–27]. It has been implied that vitamin D level is negatively associated with insulin resistance, glucose intolerance and obesity [6, 24, 26]. Meanwhile, clinical trials have also suggested an inverse link between vitamin D concentration and type II diabetes [6, 25]. In the experimental mice with 1α(OH)ase deficiency, insulin resistance and hyperglycemia were also developed [28, 29]. More importantly, exogenous supplementation of active vitamin D3 could decrease the incidence of insulin resistance [6, 24–27]. Thus, vitamin D deficiency could be associated with insulin resistance and type II diabetes, yet the underlying mechanisms are largely unknown [6, 24–27]. In the current study, we showed that vitamin D deficiency via stably silencing 1α (OH)ase also caused insulin resistance in L02 hepatocytes, showing impaired insulin signaling and downregulation of GLUT4.

It has been proposed that vitamin D could increase the release of some anti-inflammatory cytokines, whiling decreasing the production of some pro-inflammatory cytokines [25, 30, 31]. Other studies, however, showed that vitamin D supplementation in humans showed no beneficial effect on inflammation [6]. Existing studies have also proposed vitamin D, especially its active D3 form, as an effective antioxidant [17, 18, 32, 33]. Systemic administration of vitamin D3 attenuated iron-induced oxidative damage in brains [17, 18, 32, 33]. Vitamin D3 could promote expression of several anti-oxidative genes, including γ-glutamyl transpeptidase (GGT), glutathione, glutathione peroxidase (GPx), and SOD, among others [17, 18, 32, 33]. On the other hand, vitamin D-deficiency would lead to oxidative stresses [17, 18, 32, 33].

In the current study, we proposed that oxidative stress could be the main reason of insulin resistance in vitamin D-deficient cells. Vitamin D depletion via stably silencing 1α (OH)ase in L02 hepatocytes led to significant ROS production, as well as subsequent p53-p21 activation and DNA damage. Such effects were almost completely nullified with co-treatment of NAC, the known anti-oxidant. Remarkably, insulin resistance was also abolished with co-treatment of NAC in 1α(OH)ase-depleted L02 hepatocytes. Together, our results suggest that vitamin D deficiency-induced insulin resistance is possibly caused by oxidative stress in L02 hepatocytes.

## MATERIALS AND METHODS

### Chemicals and reagents

Insulin, n-acetylcysteine (NAC) and puromycin were provided by Sigma Aldrich Chemicals (Nanjing, China). The antibodies of this study were provided by Cell Signaling Technology (Danvers, MA) and Abcam (Shanghai, China). The reagents for cell culture were obtained from Gibco Co. (Nantong, China).

### Cell culture

Human L02 hepatocytes were provided by the Cell Bank of Shanghai Biological Institute (Shanghai, China). L02 cells were cultured in RPMI 1640 with 10% FBS and antibiotics, in a humidified atmosphere at 37°C with 5% CO_2_. Cells were subjected to mycoplasma and microbial contamination examination every 2-3 months. Population doubling time, colony forming efficiency, and morphology were routinely checked to confirm the genotype.

### 1α(OH)ase shRNA

The two lentiviral 1α(OH)ase shRNAs with non-overlapping sequences were designed and verified by Genepharm (Shanghai, China). The shRNA (10 μL/mL medium, per well) was added to L02 hepatocytes for 24 hours. Stable cells were selected by puromycin (0.5 μg/mL, Sigma) for a total of 10 days. Puromycin-containing medium was renewed every two days. Silence of 1α(OH)ase in the stable L02 hepatocytes was confirmed by both qRT-PCR assay and Western blotting assay. Control cells were infected with lentiviral scramble control shRNA (Santa Cruz Biotech).

### Vitamin D3 assay

The level of vitamin D3 in the conditional medium of L02 hepatocytes were tested via a commercial available enzyme linked fluorescent assay (ELFA) kit from Roche (Shanghai, China), according to the manufacturer's guidelines.

### RNA isolation and qRT-PCR

As described in our previous studies [34, 35], Trizol reagents (Invitrogen) was applied to extract the total RNA of L02 hepatocytes. RNA was then reverse-transcribed (RT) with RT-PCR kit (Toyobo, Osaka, Japan). Quantitative Real-time PCR (“qRT-PCR”) assay using the SYBR green kit was performed using the ABI-7600 PCR system (Applied Biosystems, Shanghai, China). mRNA primers of *1α(OH)ase* and GAPDH were described previously [36, 37]. We utilized the 2^ΔΔCt^ method to calculate relative *1α(OH)ase* mRNA expression (vs. GAPDH). All the primers were synthesized by Genepharm (Shanghai, China).

### Western blotting assay

First, the lysis buffer (Biyuntian, Wuxi, China) was applied to achieve protein lysates from L02 hepatocytes. For each condition, 30 μg total lysate proteins per lane were separated by the SDS-PAGE gels (10-12%) [16, 38], which were then transferred to the polyvinylidene difluoride (PVDF, Millipore, Suzhou, China) membranes. Afterwards, the blots were blocked (in 10% of milk), and were incubated with designated primary and corresponding secondary antibodies. Enhanced chemiluminescence (ECL) reagents (Pierce, Nantong, China) were utilized to visual the interested bands [39–41]. Total gray of each band was quantified via the ImageJ software, and the value was normalized to that of loading control (ERK1/2 or β-Tubulin).

### SOD activity assay

The superoxide dismutase (SOD) activity in L02 hepatocytes was assayed by the NWLSS kit, which is an extremely sensitive SOD kit, using WST-1 to generate a water-soluble formazan dye upon reduction with superoxide anion. The detailed procedure was described previously [42]. The final mixture was subjected to spectrophotometer detection at the absorbance at 560 nm.

### Reactive oxygen species (ROS) assay

ROS content was tested by the DCFH-DA fluorescent dye assay (Invitrogen). The detailed protocol was described in our previous studies [34, 35, 41]. Briefly, after applied treatment, L02 hepatocytes were incubated with 10 μM of DCFH-DA for 30 min under the dark, which were thereafter tested for fluorescence under a Fluorescence Microplate Reader (Synergy 2, BioTek, Winooski, VT).

### Lipid peroxidation assay

As described previously [43], cellular lipid peroxidation was evaluated by the thiobarbituric acid reactive substances (TBAR) assay [44]. Briefly, after the indicated treatment, L02 hepatocytes protein lysates (20 μg per condition) were mixed with 20% of acetic acid and thiobarbituric acid solution. After heating, the mixtures were centrifuged, and then in the supernatant the red pigment dye was tested via a microplate reader. TBAR activity was expressed as nM of malondialdehyde per mg protein. The values of treatment group were always normalized to control.

### γ-H2AX FACS assay

γ-H2AX assay was performed to test DNA damage. Briefly, L02 hepatocytes were first trypsinized and fixed in ice-cold ethanol, which were then exposed to the anti-γ-H2AX antibody (Cellular Signaling Tech) for 6 hours. Cells were then incubated with the FITC-conjugated secondary antibody. FACS assay was performed to determine γ-H2AX percentage, as the quantitative measurement of DNA damage [45].

### Statistical analysis

The results were expressed as mean ± standard deviation (SD). The statistical analysis among different groups was done using one-way ANOVA with Scheffe's test [46, 47].
